# Umbelliferone Ameliorates Memory Impairment and Enhances Hippocampal Synaptic Plasticity in Scopolamine-Induced Rat Model

**DOI:** 10.3390/nu15102351

**Published:** 2023-05-17

**Authors:** Ga-Young Choi, Hyun-Bum Kim, Jae-Min Cho, Inturu Sreelatha, In-Seo Lee, Hee-Seok Kweon, Sehyun Sul, Sun Ae Kim, Sungho Maeng, Ji-Ho Park

**Affiliations:** 1 Center for Research Equipment, Korea Basic Science Institute, Cheongju 28119, Republic of Korea; 2 Department of Systems Pharmacology and Translational Therapeutics, Perelman School of Medicine, University of Pennsylvania, Philadelphia, PA 19104, USA; 3 Graduate School of Biotechnology, Kyung Hee University, Yongin 17104, Republic of Korea; 4 Department of Gerontology (AgeTech Service Convergence Major), Graduate School of East-West Medical Science, Kyung Hee University, Yongin 17104, Republic of Korea; 5 Undergraduate Programs, Rutgers University, 100 Rockafeller Road, Suite 1008, Piscataway, NJ 08854, USA

**Keywords:** Alzheimer’s disease, umbelliferone, scopolamine, long-term potentiation, memory, hippocampus

## Abstract

Alzheimer’s disease (AD) is a neurodegenerative disorder, characterized by memory loss and cognitive decline. Among the suggested pathogenic mechanisms of AD, the cholinergic hypothesis proposes that AD symptoms are a result of reduced synthesis of acetylcholine (ACh). A non-selective antagonist of the muscarinic ACh receptor, scopolamine (SCOP) induced cognitive impairment in rodents. Umbelliferone (UMB) is a Apiaceae-family-derived 7-hydeoxycoumarin known for its antioxidant, anti-tumor, anticancer, anti-inflammatory, antibacterial, antimicrobial, and antidiabetic properties. However, the effects of UMB on the electrophysiological and ultrastructure morphological aspects of learning and memory are still not well-established. Thus, we investigated the effect of UMB treatment on cognitive behaviors and used organotypic hippocampal slice cultures for long-term potentiation (LTP) and the hippocampal synaptic ultrastructure. A hippocampal tissue analysis revealed that UMB attenuated a SCOP-induced blockade of field excitatory post-synaptic potential (fEPSP) activity and ameliorated the impairment of LTP by the NMDA and AMPA receptor antagonists. UMB also enhanced the hippocampal synaptic vesicle density on the synaptic ultrastructure. Furthermore, behavioral tests on male SD rats (7–8 weeks old) using the Y-maze test, passive avoidance test (PA), and Morris water maze test (MWM) showed that UMB recovered learning and memory deficits by SCOP. These cognitive improvements were in association with the enhanced expression of BDNF, TrkB, and the pCREB/CREB ratio and the suppression of acetylcholinesterase activity. The current findings indicate that UMB may be an effective neuroprotective reagent applicable for improving learning and memory against AD.

## 1. Introduction

Neurodegenerative disorders (NDDs) are common health problems for the global aging population. Every year, the incidence of NDDs is growing [1], due to increasing life expectancy worldwide [2]. NDDs are manifested by clinical characteristics, such as extrapyramidal and pyramidal movement disorders and cognitive or behavioral disorders. Common causes of NDDs are the accumulation of specific proteins and anatomical fragility [3], such as in Alzheimer’s disease (AD), Parkinson’s disease (PD), and Huntington’s disease. Specifically, AD is characterized by progressive learning and memory impairment in association with deposition of the β-amyloid (Aβ) protein, tau neurofibrillary tangles, cholinergic nervous system disorders, neuronal apoptosis, neuroinflammation, and oxidative stress [2,4]. The cholinergic hypothesis suggests that AD is the result of the decreased synthesis of acetylcholine (ACh) due to the decreased number of neurons in the basal ganglia [1]. Learning and memory function depends on the activation of neurotransmitters, such as ACh, dopamine, and serotonin, which transmits external signals to the intercellular messengers [5]. Moreover, it has been shown that ACh plays an important role in the hippocampal mode shifting between encoding and retrieval [6].

Scopolamine (SCOP) is a non-selective post-synaptic muscarinic ACh receptors antagonist [7] and has been used as a standard/reference drug in the field of neuropsychopharmacology to induce impairments of learning and memory in animals models [6,7,8]. Previous studies showed that SCOP can disrupt short-term or long-term spatial, fear avoiding, and working memory [9,10,11]. Acetylcholinesterase inhibitors (AChEIs), such as donepezil, were developed for the relief of AD symptoms [12], but, due to adverse effects including depression, anorexia, nausea, and drowsiness, the efficacy is not clear [13]. Therefore, to develop a safe and effective AD treatment is an urgent requirement. Numerous pharmaceutical institutes tried to develop health supplements to improve AD symptoms based on herb-derived compounds [14,15].

Umbelliferone (UMB) is a coumarin compound distributed in the Apiaceae family of plants such as carrot, coriander, and wild celery [4,16]. UMB is known for its antioxidant, anti-inflammatory, anticancer, antimicrobial, antibacterial, and antidiabetic properties [17,18]. UMB was also reported to have neuroprotective effects in rat models of chronic unpredictable mild stress-induced depression [19,20], sporadic AD [21], PD [22], and focal cerebral ischemia–reperfusion [23]. However, the effect of UMB on ACh-related learning and memory deficit is not yet clearly reported.

Brain-derived neurotrophic factor (BDNF), a well-known neurotrophic protein of the nervous system, is involved in neuronal survival, growth, differentiation, and maturation [24,25] through the tropomyosin receptor kinase B (TrkB) receptor binding and subsequent CREB phosphorylation [26,27,28,29]. The BDNF/TrkB/CREB pathway is known to be involved in antidepressant, anti-apoptotic, and antioxidative mechanisms [30,31,32]. Moreover, this pathway regulates the proliferation, differentiation, and migration of hippocampal neurons, which are important for cognitive functions [33]. In this study, it was hypothesized that UMB may enhance learning and memory via the BDNF/TrkB/CREB pathway.

Long-term potentiation (LTP) is the key process of synaptic plasticity in association with a continuous increase in synaptic strength [34,35]. LTP is a mechanism for information storage, which is the basis for the behavioral and psychological phenomena in learning and memory formation [36]. LTP can be induced by the activation of the NMDA (N-methyl-D-aspartate) and AMPA (α-amino-3-hydroxy-5-methyl-4-isoxazolepropionic acid) glutamate receptors [37]. In the LTP between CA3-CA1 Schaffer collateral synapses, the AMPA receptor is a driving force behind glutamatergic transmission and induces large and rapid synaptic signaling [38]. Some studies showed that SCOP impaired LTP responses, which demonstrates the involvement of cholinergic drives depending on the activation of muscarinic receptors upon LTP induction in the hippocampus [39,40].

Synapses are the structural and functional connection between neurons, providing the biological basis of learning and memory. Synaptic plasticity is crucial in turning temporary memory into permanent memory and is related to LTP [41]. Studies showed that the density of synaptic vesicles plays a central role in the regulation of synaptic plasticity, which is involved in the storage, uptake, and stimulus-dependent release of neurotransmitters [41,42].

In this study, the effects of UMB on learning and memory impairment and its related molecular mechanisms are investigated. We found that UMB can ameliorate learning and memory deficits in the SCOP-induced animal model through the activation of the BDNF/TrkB/CREB pathway. Behavioral measurements demonstrated improvements in learning and memory. Moreover, UMB had a synaptic strengthening effect on the modification of the synaptic ultrastructure in association with NMDA/AMPA receptors.

## 2. Materials and Methods

### 2.1. Materials

Umbelliferone (H24003), SCOP (S0929), penicillin streptomycin (P4333), HPEPS [4-(2-hydroxyethyl)-1-piperazineethanesulfonic acid, H4034], dimethyl sulfoxide (237329), l-glutamine (G-8540), and d-glucose (G-7528) were obtained from Sigma-Aldrich (St. Louis, MO, USA). MEM (Minimum Essential Medium, LM 007-01) and HBSS (Hank’s balanced salt solution, LB 003-01) were purchased from Welgene (Kyungsan, Republic of Korea). Heat-inactivated donor horse serum (HS, S0900-500) was supplied by Biowest (Nuaillé, France). Protease inhibitors were purchased from GenDEPOT (Katy, TX, USA). Lysis buffer was obtained from ByLabs (Namyangju, Republic of Korea).

### 2.2. Animals

Adult male SD (Sprague-Dawley) rats (weighing 190–230 g; 7~8 weeks old) were purchased from Saerone Bio (Uiwang, Republic of Korea). During the experiment, rats were maintained under 12 h light/dark cycle in a facility with a regulated temperature (23 ± 1 °C), humidity (50–60%), and standard diet, and water was provided ad libitum. The rats were acclimated to the new environment for 5 days before the experiments. Next, rats were randomly divided into 4 groups: the control group, the UMB group, the SCOP group, and the SCOP + UMB group. All experiments were conducted in accordance to the National Institutes of Health’s Guide for Care and Use of Laboratory Animals (2011) after the approval of the Institutional Care and Use Committee of Kyung-Hee University (No. KHGASP-22-052). All attempts were made to minimize the suffering of animals and limit their sacrifice.

### 2.3. Experimental Design

**Figure 1:**
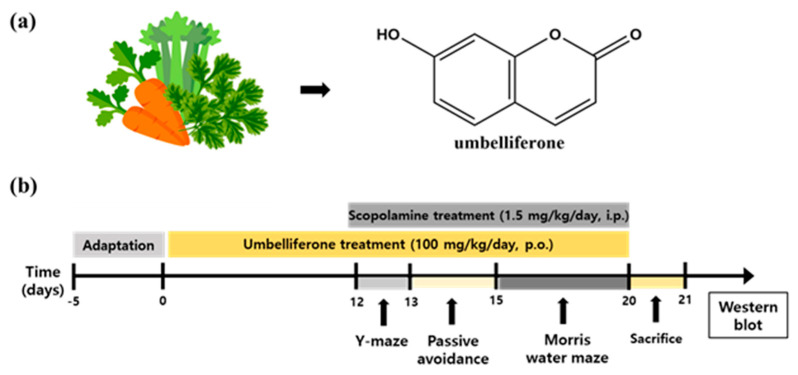
The chemical structure of UMB and experiment timeline. (**a**) The chemical structure of UMB. UMB is derived from carrot, coriander, and wild celery. (**b**) Experimental timeline of drug treatments and behavioral tests. All rats were treated with drugs after the adaptation period. Control group: PBS (1 mL/day, p.o. and 1 mL/day, i.p.); UMB group: UMB (100 mg/kg/day, p.o.) and PBS (1 mL/day, i.p.); SCOP group: PBS (1 mL/day, p.o.) and SCOP (1.5 mg/kg/day, i.p.); UMB + SCOP group: UMB (100 mg/kg/day, p.o.) and SCOP (1.5 mg/kg/day, i.p.). Behavioral tests were commenced on day 12, and the rats were sacrificed for tissue collection on day 20.

UMB and SCOP were dissolved in PBS (phosphate-buffered saline, pH 7.4). UMB was administered at a dose of 100 mg/kg/day per oral (p.o.) for 21 days, and SCOP was injected with a dose of 1.5 mg/kg/day by intraperitoneal (i.p.) for 10 days. The dose of UMB was determined based on the previously published literature [43,44]. Dosing solutions were freshly prepared every day. The concentration and administration period of SCOP were determined according to a previous study [40]. The control group received PBS 1 mL/day, p.o. and PBS 1 mL/day, i.p. The UMB group received UMB 100 mg/kg/day, p.o. and PBS 1 mL/day, i.p. The SCOP group was administered with PBS 1 mL/day, p.o. and SCOP 1.5 mg/kg/day, i.p. The UMB + SCOP group was administered with UMB 100 mg/kg/day, p.o. and SCOP 1.5 mg/kg/day, i.p. The UMB treatment was maintained from the beginning to the end of the experiment. The SCOP treatment was administered for the eight days during the behavioral tests. UMB was treated for 60 min, and SCOP was treated for 30 min before each test. All rats were sacrificed for tissue extraction after all behavioral experiments were completed (Figure 1).

### 2.4. Y-Maze Test

**Figure 2:**
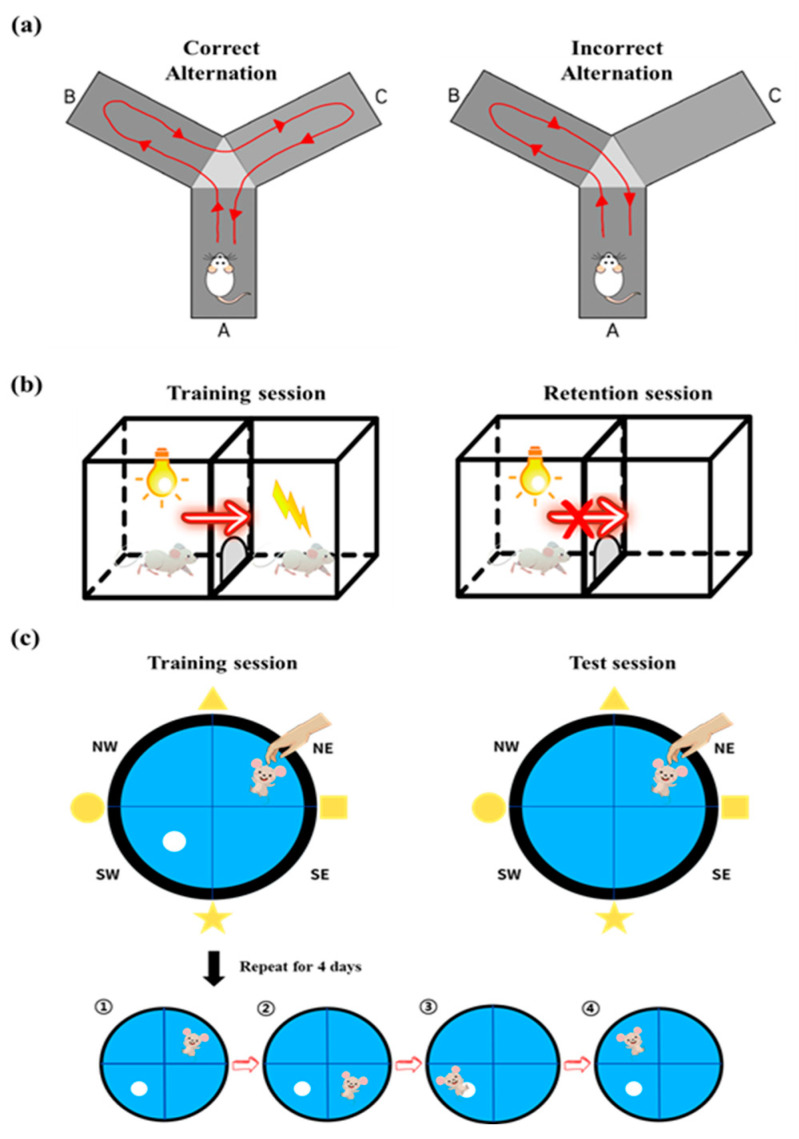
The illustrations of behavioral tests and timeline. (**a**) Y-maze test. Spontaneous alternation was defined as ‘correct’ if the rat visited the three arms consecutively. Visiting any individual arm more than once in three alternations was defined as ‘incorrect’. A, B, C represent different arms in the maze. (**b**) Passive avoidance test. In the training session, when the rat entered the dark compartment, an electrical shock was delivered to the paws. In the retention session, the latency to enter the dark compartment was measured. (**c**) Morris water maze test. During training sessions, rats were given four trials for 4 days to locate the submerged platform using visual cues starting at different locations as denoted 1, 2, 3, 4. During the test session, rats were allowed to freely navigate in the pool, with the platform removed.

For the assessment of the willingness of rodents to explore a novel place, the Y-maze test was carried out on the 12th day of UMB administration. The rats were administered UMB for 60 min and SCOP for 30 min before the test. The Y-maze was constructed with three arms (45 cm length × 35 cm height × 10 cm width, intersecting 120° apart). The initial arm closest to the experimenter was labeled as B, and the other two arms were labeled A and C. Rats was placed in the center of the Y-maze and allowed to explore for 10 min, and the results were analyzed with a videotracking system (SMART v3.0, Panlab Harvard Apparatus, Barcelona, Spain). The rats were considered to have entered the arm if all four of their paws were inside. Spontaneous alternation was defined as ‘correct’ if the rat visited the three arms consecutively. Meanwhile, visiting any individual arm more than once in three alternations was defined as ‘incorrect’ (Figure 2a). The percentage of spontaneous alternation was calculated as follows: [(number of successful spontaneous alternations)/(total number of arm entries − 2)] × 100 [45].

### 2.5. Passive Avoidance Test

The passive avoidance (PA) test was used to evaluate avoidance memory [46]. The protocol was slightly modified based on our previous study [40]. The apparatus consisted of a lit compartment (17 × 12 × 15 cm) and a dark compartment of the same size divided by an automatic guillotine door (9 × 17 cm) that can be opened to allow passage. The floor was constructed out of stainless steel with a diameter of 2.5 mm and was connected to a stimulator, which delivered an electric shock on the grid floor of the dark compartment. The experiment progressed to two independent trials: a training session for the acquisition of fear and a retention session to determine whether the fear memory remained. Each rat was apparatus during the training session. The rat was initially placed in the light compartment, facing away from the middle door, and the door was automatically opened 60 s later. When all four paws of the rat entered the dark compartment, the door was closed automatically, and the rat immediately obtained an electrical shock (1.5 mA, 2 s) to the paws. The rat was taken from the dark compartment and returned to the cage after 20 s. The time taken (latency) for the rat to enter the dark compartment was recorded. The retention session was conducted 24 h following the training session. The rat was placed in the light compartment for 60 s, as in the training, and the latency time to enter the dark compartment was measured up to a maximum of 600 s (Figure 2b). If the rat entered the dark compartment, it meant that the memory of fear had disappeared. If not, the fear of electric shock was successfully switched to memory [47].

### 2.6. Morris Water Maze Test

The Morris water maze (MWM) test is aimed at assessing long-term spatial learning and memory by measuring the latency to find a submerged escape platform in opaque water using distal cues. The MWM testing protocol was revised from a previous manuscript [48]. A 180 cm × 45 cm (diameter × height) round pool was filled with opaque water by adding liquid tempura white paint to a depth of 35 at 25 ± 1 °C. Four different-shaped visual cues were assigned to the four different directions of the maze. An escape platform was submerged 1 cm below the water surface. The rats were trained four times a day for four days, each time starting at four different quadrant area. If rats reached the platform within 60 s, they were permitted to stay on the platform for 20 s and then were transferred to their home cage. If a rat failed to locate the submerged platform within 60 s, the experimenter directed the rat to the platform. The latency time to reach the submerged platform was recorded. The probe test was performed on the fifth day. Rats were permitted to navigate freely without the platform for 90 s (Figure 2c). All movements were recorded with a video camera (SHC-650A; Samsung, Suwon, Republic of Korea). Traveling pathway, latency to the platform, and remaining time in the target quadrant were analyzed using SMART video monitoring software (SMART v3.0, Panlab Harvard Apparatus, Barcelona, Spain).

### 2.7. Western Blot Analysis

Rats were euthanized on the 21st day, and the brain was dissected out for tissue collection. Extracted hippocampus was immediately stored at −80 °C until use. The frozen hippocampus was homogenized in lysis buffer containing protease inhibitor cocktail (P3200; GenDEPOT, Katy, TX, USA) on ice for 30 min. The supernatant was obtained by centrifugation at 28,487× *g* at 4 °C for 20 min. The protein mass (17.60 ± 0.30 μg) of the supernatant was measured by the Bradford protein assay [49]. For Western blot analysis, equal quantities of hippocampal protein mass were separated by being electro-transferred onto polyvinylidene fluoride (PVDF) membranes and 10% sodium dodecyl sulfate-polyacrylamide (SDS) gel electrophoresis. The membranes were incubated in TBS(tris-buffered saline) containing 0.1 % Tween 20 (TBST) and 5% dried skim milk for 1 h and incubated overnight at 4 °C in TBST containing 5% dried skim milk and primary antibodies. Primary antibodies used were 1:2000 dilution of rabbit monoclonal antibodies against cAMP response element-binding protein (ab32515; abcam, Cambridge, UK), brain-derived neurotrophic factor (BDNF) (ab108319, abcam,), polyclonal antibody against tropomyosin receptor kinase B (TrkB) (ab18987, abcam), and glyceraldehyde 3-phosphate dehydrogenase (GAPDH) (14C10; Cell Signaling Technology, Danvers, MA, USA). Following three washes with TBST, the membranes were incubated with a 1:2500 dilution of anti-rabbit IgG horseradish peroxidase-linked antibody for a secondary antibody (7074S, Cell Signaling Technology, Danvers, MA, USA) in TBST with 5% skim milk at room temperature for 1 hr. After washing, protein bands were visualized using enhanced chemiluminescence (ECL) solution (EzWestLumi plus, WSE-7120S, ATTO Co., Tokyo, Japan) and captured on the ECL Western Blotting Detection System (ATTO Co., Tokyo, Japan). Band density was quantified using ImageJ 1.53p software (NIH, Bethesda, MA, USA).

### 2.8. Measurement of Achtylcholinestarase (AChE) Activity

The activity of AChE was carried out using a colorimetric AChE assay kit from Abcam (Cambridge, UK, a138871). First, 0.2 U/mL of AChE and the reaction mixture were applied to each well of the 96-well microplate containing the standard and test samples. Next, the plate was protected from light exposure and incubated at room temperature for 30 min. The absorbance was scanned with a microplate reader at 410 ± 5 nm (Berthold Technologies, Bad Wildbad, Germany). Corrections were made to the measured values based on the absorbance of the reaction mixture and test samples, and the AChE activity rate was calculated as a percentage.

### 2.9. Organotypic Hippocampal Slice Cultures

Interface organotypic hippocampal slice cultures (OHSCs) were prepared as described in previous studies [50] using hippocampal slices of male SD rats purchased from Saerone Bio Inc. (Uiwang, Republic of Korea). After decapitating the rats, the brain was promptly removed and submerged in a cold HBSS solution containing 20 mM HEPES. Thereafter, the hippocampus of each temporal lobe was extracted and sliced into 350 µm sections using a tissue chopper (Mickle Laboratory Engineering Co., Gomshall, UK). Six tissue slices were attached to a 0.4 µm membrane culture insert (Millicell-CM; Merck Millipore, Bedford, MA, USA) in each well of a six-well plate. Each well was filled with 1 mL of culture medium (HBSS 25% *v*/*v* + MEM 50% *v*/*v* + HS 25% *v*/*v*, supplemented with 1 mM l-glutamine, 20 mM HEPES, 5.25 g/L d-glucose, and 1% *v*/*v* penicillin-streptomycin; pH 7.1), and the medium was replaced every two days. The culture slices were incubated for 14 days at 35 °C in an incubator with 5% CO_2_ until being used in the experiments.

### 2.10. Preparation of Organotypic Hippocampal Slice Tissue on Multi-Electrode Array (MEA) Probes

A slice of stabilized hippocampal tissue was taken from the membrane culture insert and immediately placed in aSSF (artificial cerebrospinal fluid; 114 mM NaCl, 2.5 mM NaHCO_3_, 3 mM KCl, 1.1 mM NaH_2_PO_4_, 25 mM d-glucose, 1.3 mM MgCl_2_, 2 mM CaCl_2_, and 20 mM HEPES; pH 7.4). The hippocampal slice was then put on an 8 × 8 microelectrode array (MEA) with 10 µm diameter electrodes located at 100 µm intervals and coated with 0.01% polyethylenimine. After placing the hippocampal slice, aCSF was removed, and the slice was then transferred to the MEA 1060 amplifier interface. The MEA system includes a high-density electrode array (60MEA200/30iRr-ITO, Multi-Channel Systems GmbH, Reutlingen, Germany), a stimulator (STG1004, Multi-Channel Systems GmbH), an amplifier (MEA1060, Multi-Channel Systems GmbH), and MC Rack (ver. 3.2.1.0) software for data collection (www.multichannelsystems.com (accessed on 1 August 2021)).

### 2.11. Induction of Long-Term Potentiation (LTP) in Hippocampal Slices

The point of inducing the Schaffer collateral and commissural pathway was determined based on morphological examination of hippocampus tissue and proper response to bipolar electrical stimulation. As a result, it was applied to the CA 2 region of stratum radiatum. Bipolar stimulation was set to an intensity of 160 μA and was delivered for 240 μs, and the optimal level was required to elicit 40–65% of the maximal hippocampus tissue reaction. Theta burst stimulation was comprised of 300 biphasic pulses applied in three trains of 100 Hz for 1 s at intervals of 5 min. Each experiment of LTP induction was performed for a total of 90 min, including 30 min of field excitatory postsynaptic potential (fEPSP) recordings, 10 min of theta burst stimulation, and 50 min of post-theta burst stimulation fEPSP measurements every min. During the experiment, the slices were continuously infused with aCSF bubbled with 95% O_2_ and 5% CO_2_ at a rate of 3 mL/min and were treated with control, UMB (1, 10, 100 μM), SCOP (300 μM), or UMB + SCOP (UMB (10 μM) + SCOP (300 μM)) starting 10 min after the start of recording. In addition, in the experiments of NMDA and AMPA receptors antagonists, the slices were infused with fresh aCSF and treated with DL-AP5 (50 μM), DL-AP5 (50 μM) + UMB (10 μM), CNQX (10 μM), and CNQX (10 μM) + UMB (10 μM) starting 10 min after the start of recording. All unfiltered data were sampled at 25 kHz from 60 recording channels using the recording system MC_Rack (v.3.2.1.0, Multi-Channel Systems).

### 2.12. Electrophysiology Data Processing

MC_Rack (v.3.2.1.0, Multi-Channel Systems) was utilized to digitize the analog MEA signal and isolate EPSPs from triggering amplitudes greater than 80 mV. As previously reported [51], a bespoke version of MATLAB (v.7.0.1, Mathworks, Inc., Natick, MA, USA) software was used to eliminate stimulus artifacts and integrate the evoked field potential trajectory.

### 2.13. Transmission Electron Microscopy (TEM)

The hippocampal tissue slices treated with the drugs were removed from the membrane culture insert and immediately fixed in 2.5% glutaraldehyde and 0.1 M cacodylate buffer (pH 7.3) for 2 h at room temperature. Following fixation, the tissue slices were treated with 1% OsO_4_ and 1.5% potassium ferrocyanide in 0.1 M cacodylate buffer (pH 7.3) for 1 h at 4 °C in the dark before being embedded in Epon 812 after dehydration in ethanol and propylene oxide serial soaking. Two days were spent polymerizing using pure Epon 812 resin at 70 °C. Ultrathin sections of 60 nm thickness were produced with an ultramicrotome (EM UC7, Leica, Austria) and collected onto 100-mesh copper grids. Subsequently, the slices were stained with 2% uranyl acetate (8 min) and lead citrate (5 min), and synaptic vesicles were observed at 120 kV using the KBSI Bio-HVEM System (JEM-1400 Plus; JEOL, Tokyo, Japan). The synapse density and the number of synaptic vesicles were measured with ImageJ (developed at the National Institute of Health).

### 2.14. Statistical Analysis

All data were shown as mean ± SEM (standard error of the mean). Statistical analysis was conducted using the IBM SPSS Statistics program (version 29.0; IBM Inc., Chicago, IL, USA). The significant differences in mean values were evaluated using one-way analysis of variance (ANOVA) and repeated measures of ANOVA with Tukey’s honest significance difference (HSD) post hoc test (*p* < 0.05). Body weight was compared by paired student *t*-test (*p* < 0.05).

## 3. Results

### 3.1. Change of Body Weight on the UMB-Treated Rats

**Figure 3:**
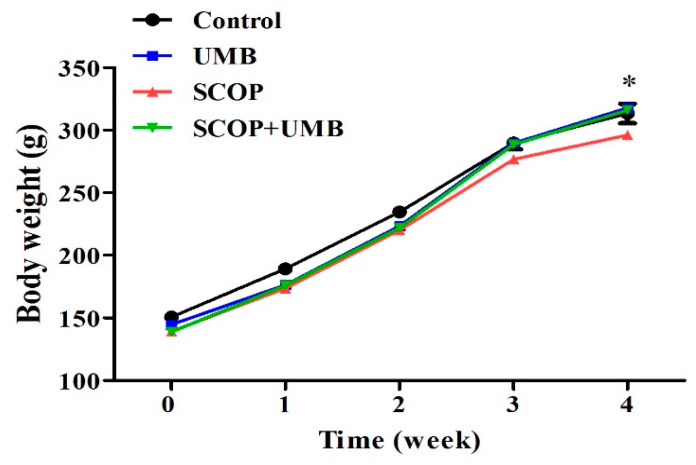
Change of body weight with UMB treatment in SCOP-induced learning and memory impairment rat model. Body weight increased in all groups during the 4-week experimental period, and the UMB-treated group gained more weight than the SCOP group. Data were represented as mean ± standard errors of the mean (SEM) (*n* = 5), * *p* < 0.05, UMB group vs. SCOP group. Analyzed by ANOVA followed by Tukey’s HSD test.

As shown in Figure 3, the average body weight was 143.35 ± 2.81 g before starting the experiment. There was no distinct difference among all groups (F(3, 16) = 2.391, *p* = 0.107). At the end of 4 weeks, all groups gained body weight (F(3, 16) = 4.311, *p* = 0.021). The body weight of the control group of animals increased to 313.40 ± 7.80 g, the body weight of the UMB group increased to 317.80 ± 7.01 g, and the body weight of the SCOP and SCOP + UMB groups increased to 296.20 ± 6.72 g and 316.20 ± 7.89 g, respectively. The UMB-treated group of animals gained more weight compared to the SCOP-treated group.

### 3.2. UMB Ameliorates Short-Term Spatial Learning and Memory Deficits in the Y-Maze Test

**Figure 4:**
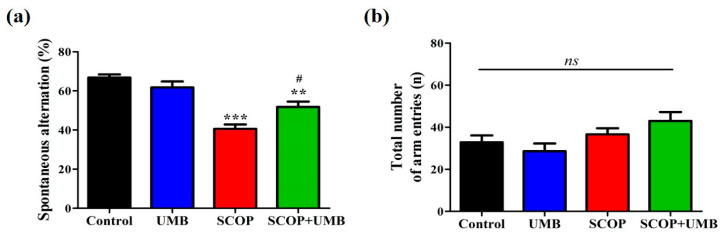
The effects of UMB on SCOP-induced spatial memory impairment in Y-maze (*n* = 5). (**a**) The percentage of spontaneous alternations in the Y-maze was calculated. (**b**) The total number of entries into three arms. There were no significant differences between groups. Data were expressed as mean ± SEM. ** *p* < 0.01, *** *p* < 0.001 vs. control group, # *p* < 0.05 vs. SCOP group, *ns*: non-significant. Analyzed by ANOVA followed by Tukey’s HSD test.

To measure the effect of UMB on spatial learning and memory impairment, the alternation was measured in the Y-maze. The results showed that the percentage of spontaneous alternation in each group was different (F(3, 16) = 21.946, *p* < 0.001, Figure 4a). The SCOP group showed a lower percentage of alternation compared to the control group (*p* < 0.001). The SCOP + UMB group showed more spontaneous alternation compared to the SCOP group (*p* < 0.05). There was no difference in the total number of arm entries (F(3, 16) = 2.939, *p* = 0.065, Figure 4b), indicating that the change in the alternation behavior had no confounding influence on locomotor activity.

### 3.3. UMB Ameliorates Fear-Avoidance Learning and Memory Deficits in the PA Test

**Figure 5:**
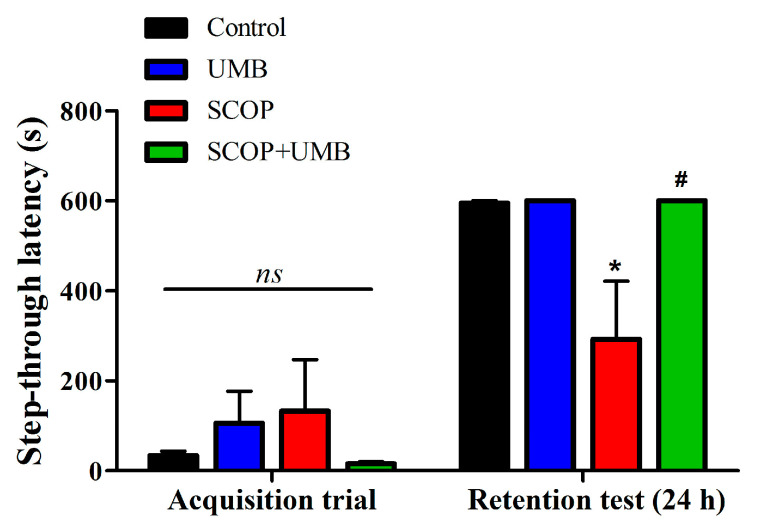
The effects of UMB on avoidance memory in SCOP-induced rats measured by the passive avoidance test. In the training session, the rats were trained to adapt to passive avoidance apparatus. When the rats entered the dark compartment, an electrical shock was given. The retention session was conducted 24 h after the training session and the latency to enter the dark compartment was measured. Data were presented as mean ± SEM, *n* = 5, * *p* < 0.05 vs. control group, ^#^ *p* < 0.05 vs. SCOP group, *ns*: non-significant. Analyzed by ANOVA followed by Tukey’s HSD test.

To examine the effect of UMB on SCOP-induced learning and memory deficit, the fear-avoidance behavior was measured (Figure 5). There were no differences in the latency to enter the dark compartment before delivering an electric shock (F(3, 16) = 0.696, *p* = 0.568). In the retention session performed 24 h after the training session (F(3, 16) = 5.565, *p* = 0.008), there was no significant difference between the control group and the UMB group (*p* = 1.000). However, the SCOP-treated group showed a substantial decrease in the step-through latency of the retention test compared to the control group (*p* < 0.05). The SCOP + UMB-treated group indicated a noticeable increase compared to the SCOP group (*p* < 0.05). This finding implies that SCOP disrupts passive avoidance memory, and UMB improves the retrieval of SCOP-induced memory damages.

### 3.4. UMB Ameliorates Long-Term Spatial Learning and Memory Deficits in the MWM Test

**Figure 6:**
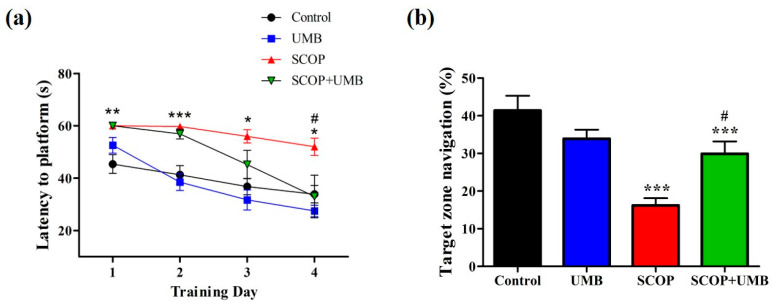
The effect of UMB on long-term spatial learning and memory as measured by the MWM test. (**a**) Escape latency (s) to the concealed platform during 4 days of training. (**b**) Percentage of time spent in the target zone during the test session. Results were provided as mean ± SEM, *n* = 5, * *p* < 0.05, ** *p* < 0.01, *** *p* < 0.001 compared with the control group; ^#^ *p* < 0.05 compared with the SCOP group. Analyzed by ANOVA followed by Tukey’s HSD test.

To assess the effect of UMB on SCOP-induced long-term spatial learning, we conducted the MWM test (Figure 6a). The escape latency gradually decreased during the training in all groups (F(3, 76) = 25.842, *p* < 0.001). The escape latency was delayed in the SCOP group compared to the control group (*p* < 0.001). However, latency in the SCOP + UMB group was shorter compared to the SCOP group (*p* < 0.001). On the 1st day, the latency of the SCOP + UMB group was the same as that of the SCOP group (60.00 ± 0.00 vs. 60.00 ± 0.00 s, respectively, *p* = 1.000). On the 4th day, however, the latency in the SCOP + UMB was shorter than that of the SCOP group (33.03 ± 8.08 vs. 52.04 ± 3.26 s, respectively, *p* < 0.05). On the 5th day, the time spent in the quadrant where the platform was previously located was measured (F(3, 16) = 19.170, *p* < 0.001; Figure 6b). The time spent in the target zone was reduced in the SCOP group in comparison to the control group (*p* < 0.001). In the SCOP + UMB group, the target zone time increased compared with the SCOP group (*p* < 0.05).

### 3.5. UMB Upregulates the Expressions of BDNF, TrkB, and p-CREB in the Hippocampus of SCOP-Induced Rat

**Figure 7:**
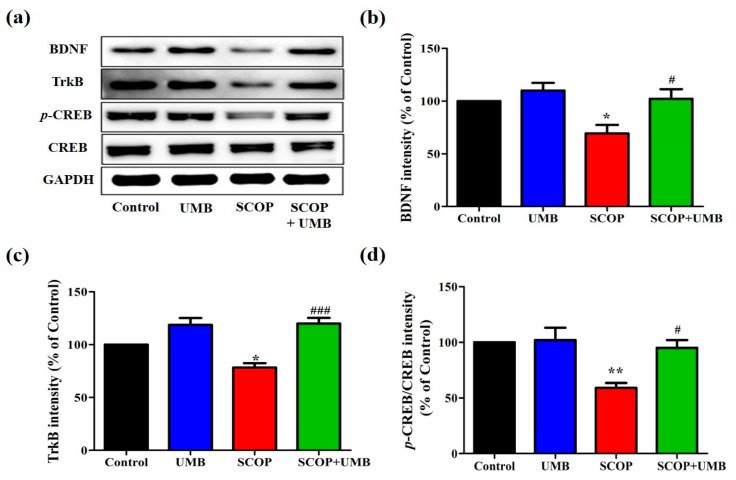
Effects of UMB on the protein expression of BDNF, TrkB, and p-CREB in the SCOP-induced rat hippocampus. (**a**) Representative images of hippocampal BDNF, TrkB, *p*-CREB, CREB, and GAPDH immunoblotting images. (**b**) The band quantification of BDNF/GAPDH ratio. (**c**) The band quantification of TrkB/GAPDH ratio. (**d**) The band quantification of (*p*-CREB/CREB)/GAPDH ratio. Data of results were shown as mean ± SEM, *n* = 5. * p < 0.05, ** p < 0.01 vs. the control group and *^#^ p* < 0.05, ^###^ *p* < 0.001 vs. the SCOP group. Analyzed by ANOVA followed by Tukey’s HSD test.

The hippocampal expression of the BDNF/TrkB/CREB signaling pathway was evaluated with immunoblotting (Figure 7a). In the expression level of BDNF (F(3, 16) = 6.301, *p* < 0.001, Figure 7b), the SCOP group showed less expression than the control group (*p* < 0.05). Meanwhile, UMB treatment improved the expression in the SCOP-treated rats (*p* < 0.05). In the expression level of TrkB (F(3, 16) = 6.933, *p* < 0.01, Figure 7c), the SCOP group showed a decrease compared to the control group (*p* < 0.05). On the other hand, the SCOP + UMB group showed an increase in the level of TrkB compared to the SCOP group (*p* < 0.001). In the case of p-CREB/CREB expression (F(3, 16) = 8.468, *p* < 0.001, Figure 7d), the levels in the SCOP group remarkably decreased compared to the control group (*p* < 0.01). However, when treated with SCOP + UMB, the protein levels of p-CREB/CREB increased compared to those of the SCOP group (*p* < 0.05). These results support the possibility that UMB treatment would improve synaptic plasticity by activating the BDNF/TrkB/CREB pathway in the hippocampus.

### 3.6. UMB Attenuates the Increase in AChE Activity Induced by SCOP in Hippocampus

**Figure 8:**
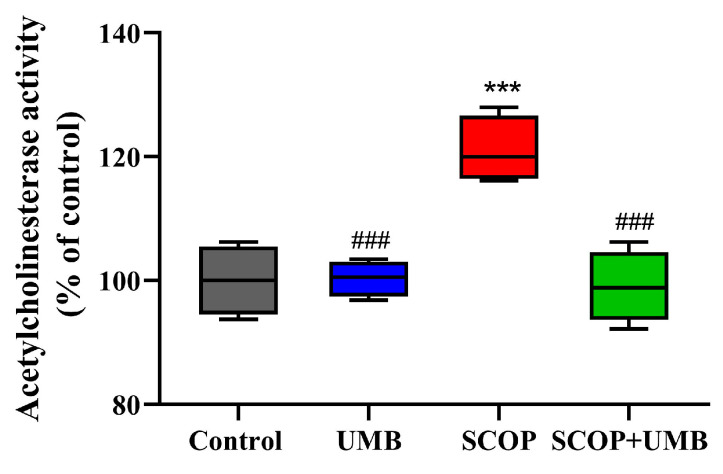
Effect of UMB on acetylcholinesterase (AChE) activity in the SCOP-induced rat hippocampus. Data were shown as mean ± SEM, *n* = 4. *** p < 0.001 vs. the control group and ^###^ *p* < 0.001 vs. the SCOP group. Analyzed by ANOVA followed by Tukey’s HSD test.

The effect of UMB on AChE activity was assessed in the hippocampus (F(3, 12) = 17.3, *p* = 0.001, Figure 8). The SCOP group showed increased activity compared to the control group (*p* < 0.001). The SCOP + UMB group showed less AChE activity compared to the SCOP group (*p* < 0.001).

### 3.7. UMB Enhances fEPSP Activity of LTP in the CA1 Region of Hippocampal Slice

**Figure 9:**
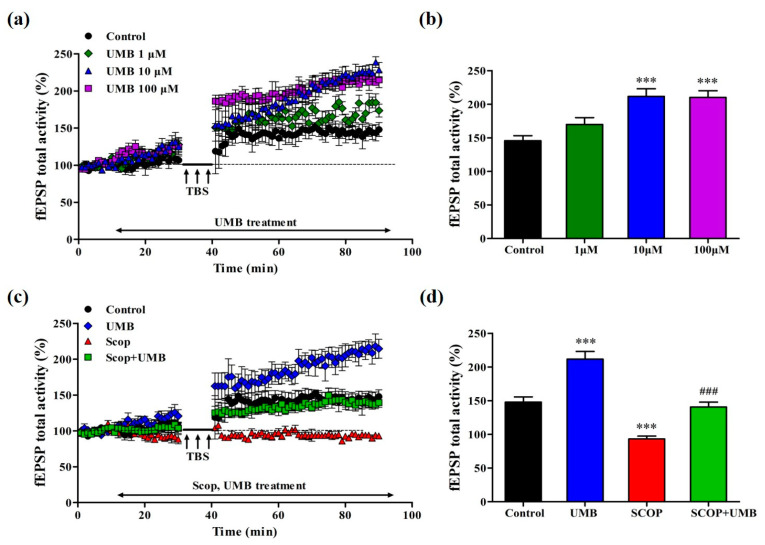
Effect of UMB on LTP in the hippocampus (*n* = 4/group). (**a**) Time course of fEPSP total activity (%) change generated by theta burst stimulation in the organotypic cultured hippocampus during the treatment of UMB. (**b**) Average total fEPSP activity from 30 to 40 min after theta burst stimulation during the dose-dependent treatment of UMB (1, 10, and 100 μM). (**c**) Time course of total fEPSP (%) activity change generated by TBS during the treatment of SCOP and SCOP + UMB in the hippocampus. (**d**) Average total fEPSP activity from 30 to 40 min after theta burst stimulation during the treatment of SCOP and SCOP + UMB. *** *p* < 0.001 vs. the control group and ^###^ *p* < 0.001 vs. the SCOP group. Analyzed by ANOVA followed by Tukey’s HSD test.

First, to test the hypothesis that UMB enhances synaptic plasticity, we investigated the effects of UMB treatment (1, 10, and 100 μM) on LTP in the hippocampal CA1 region. The time-dependent change in fEPSP activity (Figure 9a) and the mean change in fEPSP activity from 30 to 40 min after theta burst stimulation (Figure 9b) were measured (F(3, 12) = 15.112, *p* < 0.001). The total fEPSP activities of the UMB 1 μM group showed no difference compared to those of the control group. The average post-theta burst stimulation fEPSP activity from 30 to 40 min in the UMB 1 μM group was like that of the control group (*p* = 0.196). In the UMB 10 μM group, the total fEPSP activities and the average post-theta burst stimulation fEPSP activity from 30 to 40 min increased compared to those of the control group (*p* < 0.001). Moreover, compared to the control group, the total fEPSP activities and the average post-theta burst stimulation fEPSP activity from 30 to 40 min in the UMB 100 μM group increased (*p* < 0.001). As shown by the dose test, UMB enhanced the average fEPSP activity in a dosage-dependent manner.

### 3.8. UMB Ameliorates fEPSP Activity Impairment of LTP Induction by SCOP in CA1 Region of Hippocampal Slices

Next, the effect of UMB on SCOP-induced LTP impairment was examined in the SCOP and SCOP + UMB groups (Figure 9c,d). The tissues of each group were treated with SCOP 300 μM and SCOP 300 μM + UMB 10 μM. The total fEPSP activity of the SCOP group was reduced compared to that of the control group, whereas that of the SCOP + UMB group was greater than that of the SCOP group. The average post-theta burst stimulation fEPSP activity value from 30 to 40 min in each group also showed differences (F(3, 12) = 46.356, *p* < 0.001). The average post-theta burst stimulation fEPSP activity (*p* < 0.001) in the SCOP group decreased compared to that of the control group. The average post-theta burst stimulation fEPSP activity level of the SCOP + UMB group increased compared to that of the SCOP group (*p* < 0.001) and was close to the value of the control group. These results showed that UMB improved the LTP suppression induced by SCOP.

### 3.9. UMB Ameliorates fEPSP Activity Impairment of LTP by NMDA Receptor Antagonist and AMPA Receptor Antagonist in CA1 Region of Hippocampal Slices

**Figure 10:**
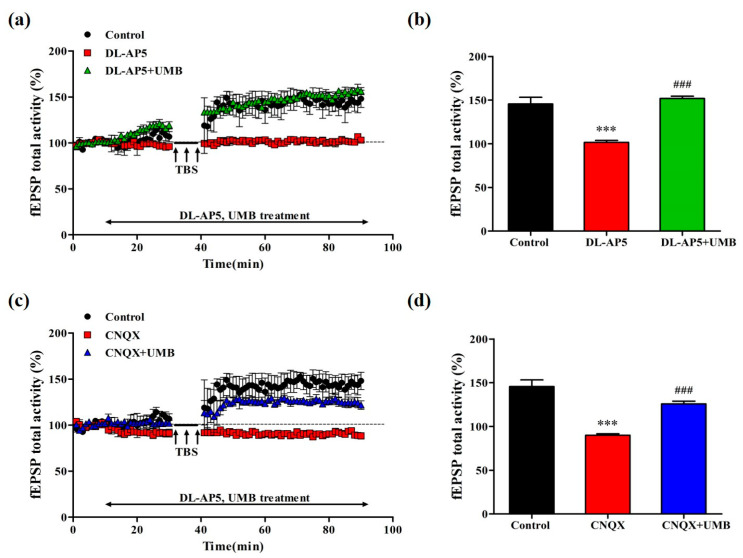
Effect of UMB on fEPSP activity in impairment of LTP by NMDA receptor antagonist and AMPA receptor antagonist in CA1 region of hippocampal slices (*n* = 4/group). (**a**) Time course of total fEPSP activity (%) change induced by theta burst stimulation in the organotypic cultured hippocampus during the treatment of DL-AP5 and DL-AP5 + UMB. (**b**) Average total fEPSP activity from 30 to 40 min after theta burst stimulation during the dose-dependent treatment of DL-AP5 and DL-AP5 + UMB. *** *p* < 0.001 vs. the control group and ^###^ *p* < 0.001 vs. the DL-AP5 group. (**c**) Time course of total fEPSP activity (%) change induced by theta burst stimulation during the treatment of CNQX and CNQX + UMB in the hippocampus. (**d**) Average total fEPSP activity from 30 to 40 min after theta burst stimulation during the treatment of CNQX and CNQX + UMB. *** *p* < 0.001 vs. the control group and ^###^ *p* < 0.001 vs. the CNQX group. Analyzed by ANOVA followed by Tukey’s HSD test.

To investigate the effect of UMB on LTP related to the NMDA receptor and AMPA receptor in the CA1 region of the hippocampus, we treated the competitive NMDA receptor antagonist, DL-AP5, and the AMPA/kainate receptor antagonist, CNQX. Previous studies reported that DL-AP5 and CNQX prevented LTP induction [52,53]. In the NMDA receptor experiment (Figure 10a,b), the tissues of each group were treated with DL-AP5 50 μM and DL-AP5 50 μM + UMB 10 μM. The total fEPSP activity of the DL-AP5 group was reduced compared to that of the control group and had similar levels to the baseline, while that of the DL-AP5 + UMB group increased compared to the DL-AP5 group, as much as the control level. The average post-theta burst stimulation fEPSP activity from 30 to 40 min in each group also showed differences (F(2, 8) = 45.561, *p* < 0.001). The DL-AP5 group decreased compared to the control group (*p* < 0.001), and the DL-AP5 + UMB group increased compared to the DL-AP5 group (*p* < 0.001), for the average post-theta burst stimulation fEPSP activity value from 30 to 40 min. In the AMPA receptor experiment (Figure 10c,d), each group of tissues was treated with CNQX 10 μM and CNQX 10 μM + UMB 10 mM (F(2, 8) = 42.941, *p* < 0.001). The total fEPSP activity of the CNQX group decreased in comparison to that of the control group; however, that of the CNQX + UMB group increased compared to that of the CNQX group. The average post-theta burst stimulation fEPSP activity values also showed that the CNQX group was lower than the control group (*p* < 0.001) and that the CNQX + UMB group was higher than the CNQX group (*p* < 0.001). The above results showed UMB ameliorated the impairment of LTP by the NMDA receptor antagonist and AMPA receptor antagonist in the CA1 region of the hippocampal slices.

### 3.10. UMB Enhanced the Synaptic Vesicle Density on Synaptic Ultrastructure of the SCOP-Treated OHSC Tissues

**Figure 11:**
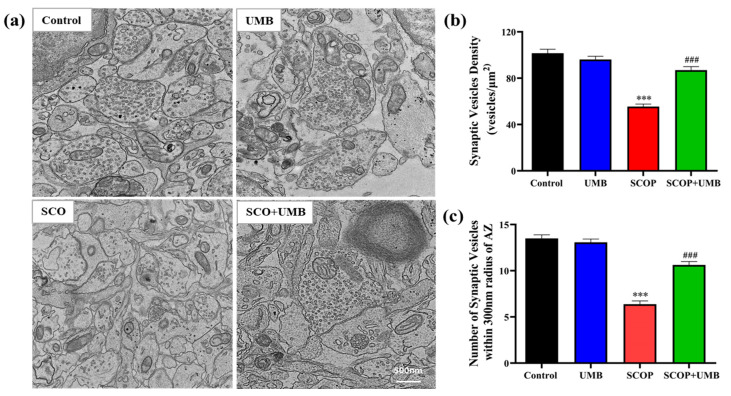
Effect of UMB on hippocampal synaptic ultrastructure of the SCOP-treated OHSC tissues. (**a**) Representative electron micrograph images of synapses in the hippocampus of each group. Scale bar = 500 nm. (**b**) Quantification of synaptic vesicle density in each group (*n* = 20). (**c**) Quantification of synaptic vesicles within 300 nm of the active zone (AZ) in each group (*n* = 40). *** *p* < 0.001 vs. the control group and ^###^ *p* < 0.001 vs. the SCOP group. Data were expressed as mean ± SEM. Analyzed by ANOVA followed by Tukey’s HSD test.

Finally, we analyzed synapses in the hippocampus treated with UMB and SCOP by quantitative electron microscopy (TEM; Figure 11a). Synapse morphology is a critical indicator for assessing synaptic plasticity, and TEM was used to observe the ultrastructure of the hippocampal synapses. In this result, the synaptic vesicle density revealed a difference among all groups (F(3, 76) = 50.4, *p* < 0.001, Figure 11b). The synaptic vesicle density rarely varied between the control and UMB groups. In contrast, the synaptic vesicle density declined in the SCOP group compared to the control group (*p* < 0.001). The SCOP + UMB group reversed the decrease in the synaptic vesicle density compared to the SCOP group (*p* < 0.001). Moreover, the number of synaptic vesicle within 300 nm of the active zone (AZ) showed a difference among all groups (F(3, 156) = 77.2, *p* < 0.001, Figure 11c). The number of synaptic vesicles within 300 nm of the AZ in the SCOP group decreased compared to the control group (*p* < 0.001). However, the number of the synaptic vesicles of the SCOP + UMB group dramatically increased above that of the SCOP group (*p* < 0.001). These results suggest that treatment with UMB enhanced synaptic plasticity by increasing the synaptic vesicle density and maintaining the synapse morphology.

## 4. Discussion

This study represented UMB’s role in the restoration of the cognitive impairments caused by SCOP through behavioral experiments and molecular biological tests. We found that UMB enhances long-term memory through electrophysiological methods. UMB increased the spontaneous alternation, step-through latency, and target zone latency, which were reduced by SCOP in the Y-maze, PA, and MWM tests. Furthermore, UMB induced the signaling molecules in the BDNF/TrkB/CREB pathway and ameliorated the fEPSP activity decreased by SCOP, DL-AP5, and CNQX.

First, we analyzed the results of behavioral tests to determine the effect of UMB on the learning and memory deficits. One of the causes of AD-related amnesia is hippocampal cholinergic dysfunction [54]. Scopolamine, as a high-affinity muscarinic acetylcholine receptor antagonist, is used for inducing hippocampal cholinergic dysfunction in terms of decreased ACh levels and muscarinic M1 receptor levels and increased AChE activity [55,56]. Previous studies reported that SCOP exerts toxicities on the dendritic development of novel hippocampal neurons, which resulted in the impairment of the circuits involved in memory processes [57]. Many behavioral experiments performed in the SCOP model showed reduced learning, memory, and cognitive abilities [40]. In this study, spontaneous alternation decreased in the SCOP group, and UMB treatment alleviated the spontaneous alternation decreased by SCOP. Moreover, there was no difference in the number of total arm entries among groups, which verified that the difference of spontaneous alternation among the groups might not be the result of changes in locomotor activity. Spontaneous alternation in the Y-maze is a behavioral test for assessing short-term spatial working memory, presenting the willingness of rodents to explore surrounding environments [58]. This test is used to assess hippocampal damage, quantify cognitive deficits, and evaluate the effects of novel drugs on cognition [59,60]. Therefore, our findings indicate that UMB ameliorates the short-term spatial working memory impaired by SCOP.

Next, the PA test was performed to measure memory based on the passive avoidance of a fear-inducing context [61]. A laboratory animal learns to avoid painful stimulus by inhibiting locomotion and exploration, which is why it is also called the inhibitory avoidance test [62]. Our results showed that the SCOP group decreased in step-through latency 24 h after the acquisition trial, indicating reduced passive avoidance memory. On the other hand, the step-through latency of animals after UMB treatment considerably increased compared to the SCOP group, which meant there was improvement in passive avoidance memory. The MWM test was used to measure long-term spatial memory and cognitive ability [63,64]. Previous studies demonstrated that UMB ameliorated cognitive deficits by enhancing learning and memory in the MWM test in streptozotocin-induced rat models of AD [21]. Hence, the present study showed that UMB co-treatment decreased the latency to the platform compared to the SCOP group during the training sessions and the time spent in the target zone of the UMB-treated group increased compared to that of the SCOP group in the test session. These results suggest that UMB can improve long-term spatial learning and memory capability.

BDNF is a neurotrophin that plays a crucial role in neural plasticity via the BDNF/TrkB/CREB pathway [26]. BDNF is abundantly expressed in the hippocampus, which is an important region of spatial learning and memory by activity-dependent synaptic plasticity with LTP [65]. It was reported that a decrease in BDNF and its receptor TrkB expression results in impaired learning and memory. BDNF and TrkB protect against memory impairment and modulate neurogenesis in the hippocampus [66]. A previous study reported that SCOP treatment decreased BDNF, p-TrkB, and p-Akt expression in the brain [67]. This study showed that the expressions of BDNF and TrkB decreased in the SCOP group, while the UMB co-treatment in SCOP-treated animals increased their expression compared to the animals only treated with SCOP. CREB is a transcription factor involved in adaptive neuronal responses as well as complex functions regulating learning and memory [68]. The activation of CREB by phosphorylation is required for long-term memory formation [69]. Furthermore, CREB phosphorylation upregulates the expression of BDNF [70]. In our study, the upregulated p-CREB/CREB ratio demonstrated the association between BDNF and CREB. Therefore, UMB could improve the learning and memory ability by protecting the neurons via the activation of the BDNF/TrkB/CREB signaling pathway.

After Bliss and Lomo published that LTP causes prolonged enhancement of synaptic transmission in 1973 [35], numerous studies have demonstrated that LTP, as a neuronal model of synaptic plasticity, contributes to the neural mechanisms underlying learning and memory [71,72,73]. In our study, the dose-dependent treatment of UMB increased the fEPSP activities of theta-burst-stimulated LTP in the hippocampus in the range from 1 to 10 to 100 μM. Especially in the UMB 10 μM group, the fEPSP activities increased more than for the other doses. According to these results, UMB can increase LTP, improve the LTP impairments induced by SCOP, and enhance synaptic strength. EPSP consists of two elements involved in the activation of different types of glutamate receptors in the hippocampus. The first one is sensitive to kainate and AMPA and related to the activation of the receptor. The second one is sensitive to NMDA [74]. NMDA receptor (NMDAR)-dependent LTPs are found in many brain regions and are mostly studied at hippocampal CA1 synapses [75]. AMPA receptors are multimeric proteins composed of GluR1, GluR2, GluR3, and GluR4 subunits that mediate the most rapid excitatory transmission in the central nervous system. Among them, the trafficking of GluR1 and GluR2 alters synaptic strength and is recognized as a central mechanism of LTP [76]. We tested the molecular identity of the UMB target during LTP induction using DL-AP5 and CNQX. DL-AP5 directly antagonizes the NMDA receptor glutamate site, and CNQX inhibits NMDA receptor activation by blocking neuronal membrane depolarization by the AMPA receptor. In this study, DL-AP5 or CNQX were co-treated with UMB on the hippocampal tissues. Both antagonists reduced the fEPSP activities of LTP. However, UMB improved the fEPSP that was reduced by these antagonists. These results indicate that UMB may affect both NMDA and AMPA receptors in relation to the LTP process.

The learning and memory impairment induced by SCOP is closely correlated with synaptic plasticity and damage, especially the morphology, interface structure, and functional status of the synapses. In previous studies, synaptic density in the synaptic morphology had sensitive characteristics and strong plasticity and was vulnerable to the afferent status of presynaptic nerve impulses as well as changes in the internal/external environment of the body [77]. According to this research, SCOP affected the synapse microstructure and caused changes such as decreased SV density and vesicles near the AZ. BDNF, which is essential for synaptic transmission and plasticity, enhances synaptic transmission, transmitter release, and the sprouting of novel synapses [78]. Mice expressing low levels of BDNF exhibited a decrease in the number of docked synaptic vesicles and showed deficiencies in synaptic sprouting [79]. Similarly, mice lacking the BDNF receptor TrkB showed a reduction in the number of docked and total synaptic vesicles and a decrease in overall synapse numbers [80,81].

Thus, the increase in SV density and the rise in levels of BDNF/TrkB/p-CREB due to the treatment of UMB led to increased synaptic plasticity. Moreover, our study suggested that UMB can possibly act as an agonist of the NMDA and AMPA receptors by opening the NMDA receptor glutamate site antagonized by AP5 and inducing depolarization of the neuronal cell membrane by CNQX to promote activation by the NMDA receptor.

## 5. Conclusions

This study revealed the effects of UMB on SCOP-induced learning and memory impairment in rats. In behavioral tests, specifically in aspects of improvement in learning and memory ability, rats treated with UMB showed improvement in cognitive ability after SCOP treatment. Moreover, UMB upregulated the expressions of factors related to the BNDF/TrkB/CREB signaling pathway and enhanced synaptic function and activity in the hippocampus through LTP and synaptic ultrastructure changes. These findings indicated that UMB could improve learning and memory by regulating the cholinergic signaling and synaptic plasticity through NMDA and AMPA receptors. Therefore, the results of the present study may be helpful for new drug discovery and in the development of therapies for neurodegenerative diseases with cognitive deficits.

## Data Availability

The data used to support the findings of this study are available from the corresponding author upon request.
